# Vestibular schwannomas and papilledema without hydrocephalus: a case report

**DOI:** 10.3389/fonc.2025.1448374

**Published:** 2025-08-19

**Authors:** Roberto Calderón-Moreno, Noé Pérez Carrillo, José de Jesús Martínez-Manrique

**Affiliations:** Unidad de Neurología y Neurocirugía, Hospital General de México, Mexico City, Mexico

**Keywords:** schwannoma, papilledema, CSF (cerebrospinal fluid), glymphatic circulation, optic nerve sheath compartment

## Abstract

Vestibular Schwannomas are frequent tumors of the cerebellopontine angle, classically presenting with cochlear and facial nerve alteration. They tend to have histopathological and intratumoral degeneration seen on MRI, and can cause CSF obstruction with hydrocephalus with subsequent visual loss. We present a case of bilateral visual loss from papilledema, with no history of hydrocephalus or increased intracranial pressure. CSF analysis showed isolated hyperproteinorrachia with no sign of infection or meningitis. This case report reviews current understanding of CSF physiology, including flow within the optic nerve sheath, which is a CSF reservoir with outflow through the complex glymphatic system. Multiple aquaporins regulate optic nerve CSF flow, responsible for the intense metabolic product washdown from the retinal apparatus. Aquaporin activity is susceptible to up-regulation or down-regulation from CSF content, such as in the complexity of certain Vestibular Schwannomas, as seen on molecular and histopathological analysis in other studies.

## Introduction

Vestibular schwannomas (VS) are tumors in the CP angle, with a reported incidence ranging from 3 to 5.2 per 100,000 people (1, 2). These tumors are classically associated with hearing loss, vertigo, and facial nerve palsy through posterior fossa cranial nerve compression and, in some cases, non-communicating hydrocephalus by obstruction of the fourth ventricle cerebrospinal fluid (CSF) flow. However, the literature suggests that the visual manifestations appear to be underreported, with decreased visual acuity seen in 8%–36% of patients across several case series, generally associated with prolonged papilledema from hydrocephalus (8). Papilledema and visual decline, however, can also occur in the absence of hydrocephalus in VS, a rare feature associated with hyperproteinorrachia by Kumar et al. in 2012 (9). In this paper, we present a case of this entity, as well as a review of the hypotheses within the study of the inflammatory microenvironment surrounding these tumors, the glymphatic system, and the CSF flow dynamics.

## Case report

**Figure 1:**
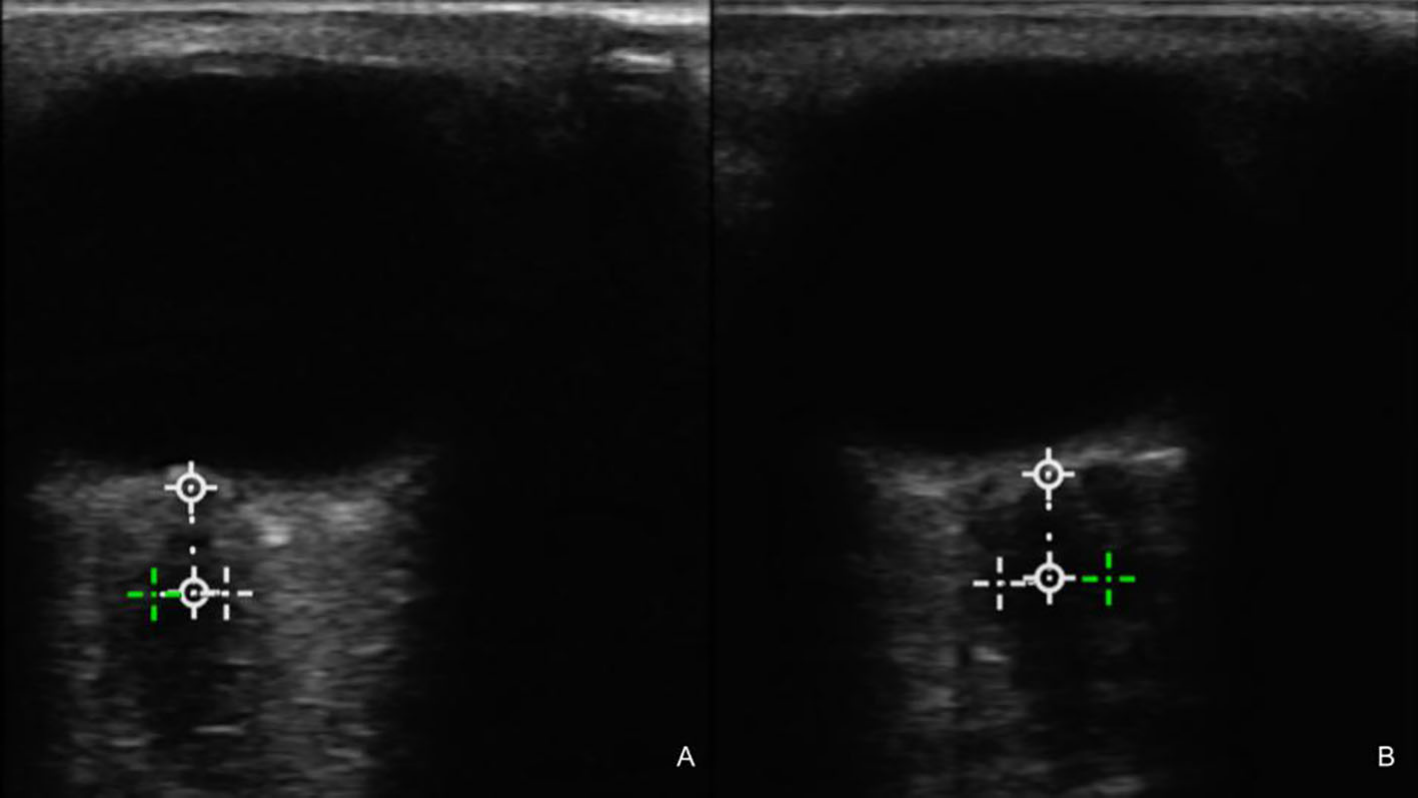
Preoperative ultrasound of the left **(A)** and right **(B)** optic nerves showing a bilateral increase in diameter. The averaged values of the optic nerve sheath diameter (ONSD) were 4.57 and 4.84 mm, respectively. The cutoff for ONSD in studies associated with elevated intracranial pressure (ICP) ranges from 4.8 to 6.4 mm (Aletreby 2022).

**Figure 2:**
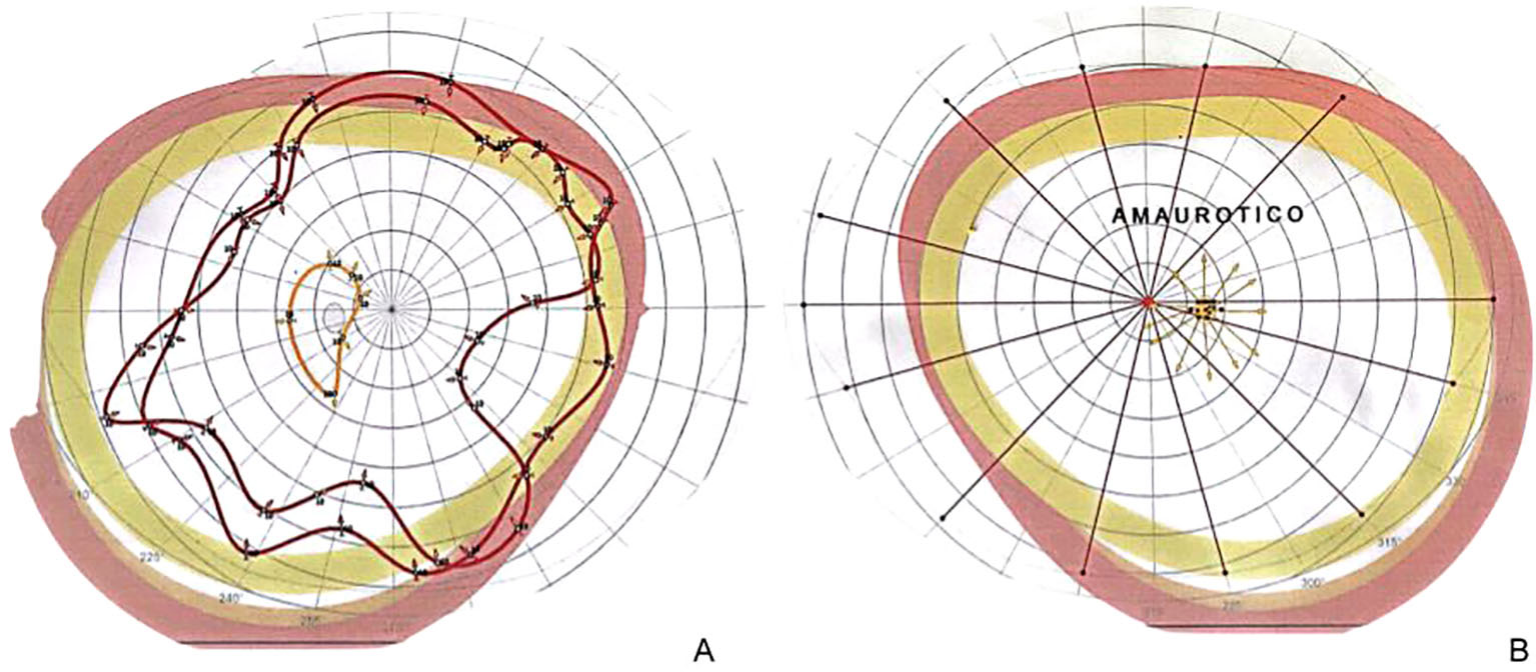
**(A)** Preoperative campimetry showing right-sided complete visual loss, reflecting optic disc swelling from sustained increased optic nerve (ON) pressure. **(B)** Left-sided campimetry notorious for an enlarged blind spot, which represents an enlarged ON disc exceedingly protruding in the posterior aspect of the optic globe, as seen on this patient’s fundus exam, as well as a peripheral scotoma.

A 23-year-old woman with no relevant prior medical history presented to the Ophthalmology Clinic of our hospital with a 12-month history of progressive bilateral visual loss. Evaluation revealed severe bilateral visual loss (no perception of light in the right eye and finger counting at 10 cm in the left eye), as well as bilateral papilledema (10) on fundus exam, which was subsequently observed by eye ultrasound (**Figure 1**). Campimetry showed right-sided amaurosis and left-sided enlarged blind spot (**Figure 2**). Upon further examination, the patient stated having continuous headaches, nausea, vertigo, left-sided hearing loss, left-sided frontal paresthesias, and right-sided lower extremity weakness for over a year. She was thus evaluated by Neurosurgery and was confirmed to have left-sided hearing loss and right-sided lower extremity weakness with hyperreflexia, with normal facial expression and cerebellar function. No diplopia was observed, and extraocular movements were preserved.

**Figure 3:**
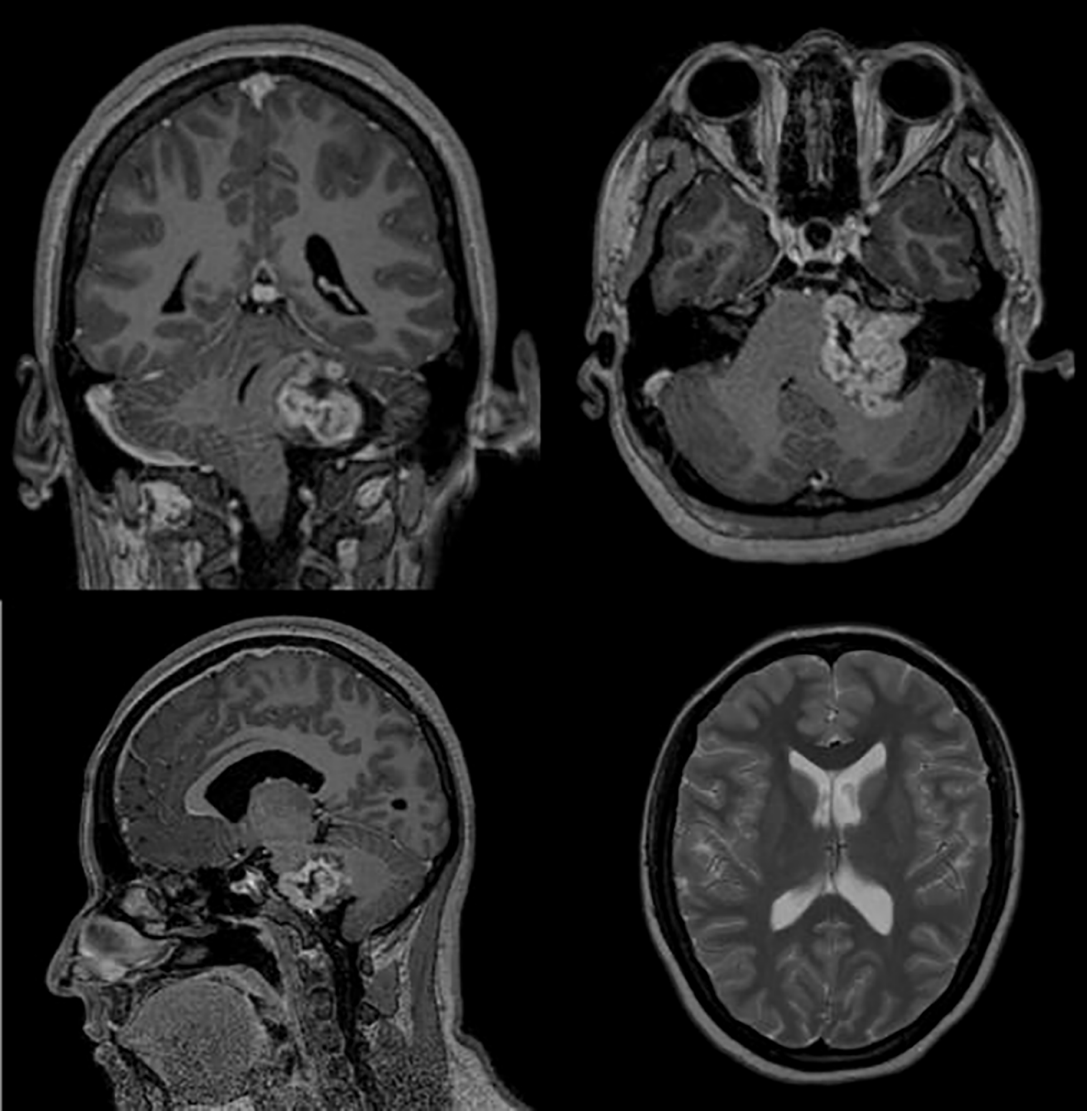
T1-weighted MRI with intravenous (IV) contrast showing a 3.4-cm × 4.4-cm × 3.3-cm contrast-enhanced lesion in the left cerebellopontine angle that extends anterolaterally into the internal auditory meatus. On the bottom right corner, a T2-weighted image shows normal lateral ventricle dimensions, demonstrating cerebrospinal fluid (CSF) system patency and absence of hydrocephalus.

**Figure 4:**
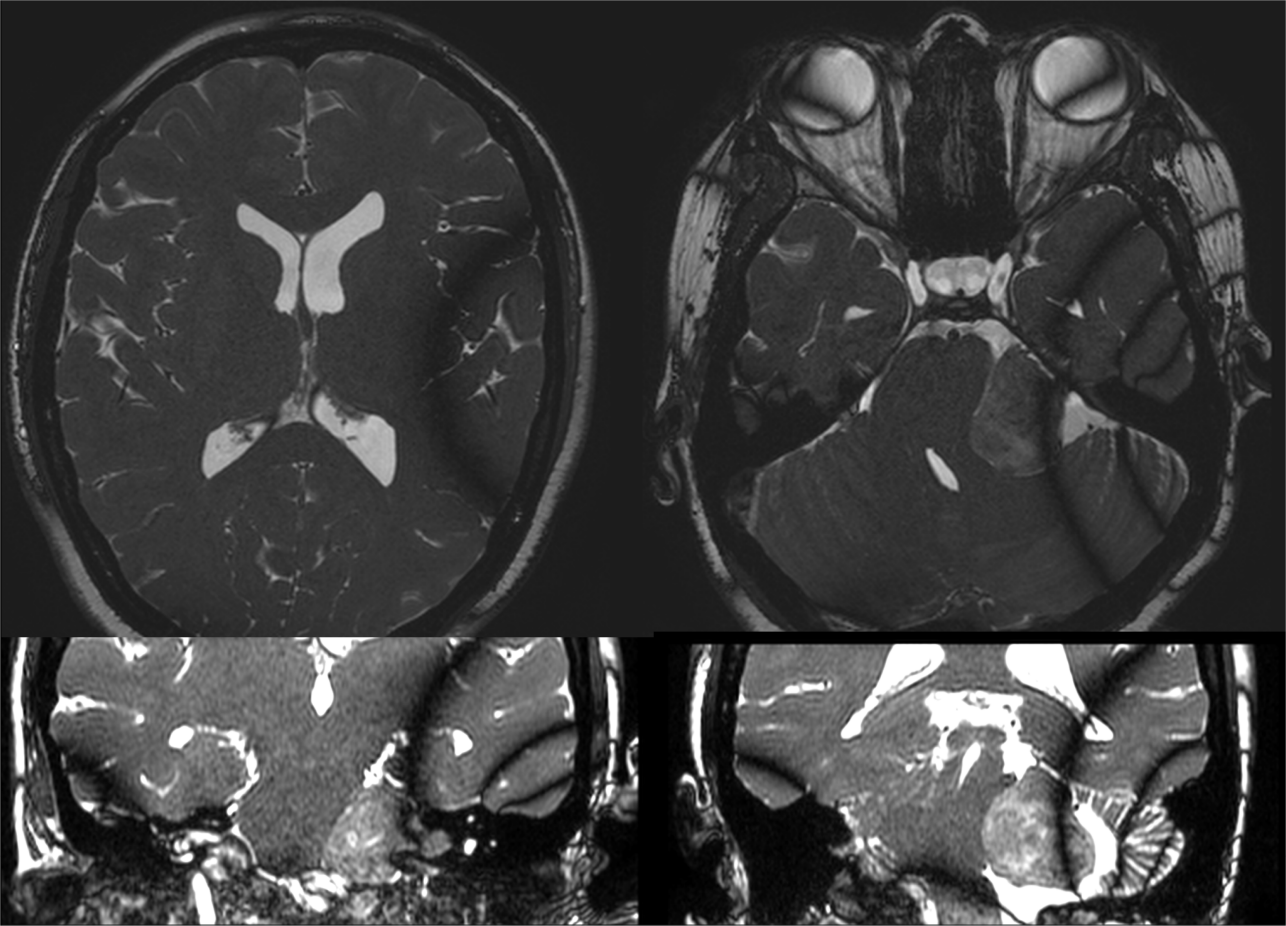
Fast imaging employing steady-state acquisition (FIESTA) T2 MRI showing no increase in lateral ventricle dimensions, third ventricle, and thin temporal horns on axial and coronal imaging.

An MRI with intravenous (IV) contrast revealed a hyperintense lesion in the left CP angle with invasion into and erosion of the internal auditory meatus, with avid contrast enhancement on T1-weighted imaging, suggestive of a left VS of Koos grade IV. No obstruction in the ventricular system was evident, nor was hydrocephalus seen on imaging, even on thin-slice T2 MRI FIESTA (fast imaging employing steady-state acquisition) sequence (**Figures 3**, **4**). No venous sinus stenosis or signs of increased intracranial pressure were found on MRI or during the clinical exam.

Considering the progressive visual decline of the patient, a prompt surgical resection was proposed. A standard retrosigmoid craniotomy in the park-bench position was performed. However, upon dural opening, immediate herniation of the left cerebellar hemisphere through the craniotomy was evident, warranting both cisterna magna drainage and craniotomy ampliation, as well as hypertonic fluid administration. Tumoral resection was postponed and the patient was admitted to Neuro-ICU, where the severe cerebellar edema without hydrocephalus was managed pharmacologically.

After 11 days of the first craniotomy, the patient was alert, with Glasgow Coma Scale (GCS) 15, no hydrocephalus, and was in good condition for surgical resection. The patient was then taken to the operating room, where the tumor was successfully resected through the previous craniotomy. The second surgery post-op went well, which included a brief overnight ICU surveillance with successful extubation, hemodynamic stability, and no neurological decline. Immediate rehabilitation consultation for facial palsy was started before discharge with in-office follow-up, and the CSF leak was successfully contained during hospitalization without the need for re-intervention. The patient had a good postoperative course and was promptly discharged.

CSF analysis of the cisterna magna fluid from the first craniotomy revealed a clear fluid with few cells and hyperproteinorrachia (312.81 mg/dl; laboratory reference value, 15–45 mg/dl) (**Table 1**). Microscopy showed no white blood cell (WBC) count abnormalities (3/mm^3^) or altered CSF glucose (57 mg/dl), but an isolated hyperproteinorrachia without signs of infection (biochemical or clinical). Gram or CSF cultures were not taken due to the low probability of an infection, besides the fact that a clear, visible cause of the altered CSF status was present. Histopathological examination confirmed the schwannoma WHO grade 1 (WHO 2021) diagnosis.

**Table 1:** Cerebrospinal fluid (CSF) cytological and cytochemical analyses obtained intraoperatively from the cisterna magna, which shows an increased protein concentration, but with the other features appearing within range.

CSF characteristics	Value	Normal range
Color/aspect	Colorless/transparent	Colorless/transparent
White blood cells/mm^3^	3 cells/mm^3^	
Red blood cells/mm^3^	6 cells/mm^3^	
Glucose	57 mg/dl	50–80 mg/dl
Proteins	312.91 mg/dl	15–45 mg/dl
LDH	11.2 IU/L	0–40 IU/L

*LDH*, lactate dehydrogenase.

Evaluation at the outpatient clinic showed a postoperative peripheral facial palsy (House–Brackmann IV) and a successfully contained CSF leak, which has been managed conservatively since. Rehabilitation for facial nerve function was started. Visual acuity did not improve. The MRI follow-up showed residual intracanalicular IV contrast-enhanced tumor, and immediate radiotherapy consultation was started for multimodality treatment. There was no residual tumor seen in the CP angle or the brainstem. The motor function of the right-sided extremities improved significantly after discharge and rehabilitation.

## Discussion

We reported on a case of VS presenting with one-sided complete visual loss confirmed by campimetry and papilledema in the absence of hydrocephalus or a clear tumoral affection to the optic nerve (ON) on MRI. In recent years, schwannomas have been regarded as increasingly complex in both histological and molecular aspects, as shown both *in vitro* and *in vivo*. The association of this feature with cisterna magna hyperproteinorrachia has been introduced more than once as either an epiphenomenon or an explanation of this complex and incapacitating pathology, although the exact mechanism remains to be elucidated.

Large studies on the clinical manifestations of VS have shown that the most frequently affected cranial nerve is the cochlear nerve (92% of schwannomas, which causes hearing loss or tinnitus), followed by the trigeminal nerve (53%). Visual manifestations are far less common, occurring in up to 16% of cases in one study, and may range from diplopia through posterior fossa ocular motor nerve affection to visual loss by compression of the optic pathway (11). Decreased visual acuity has been described in 8%–36% of schwannomas in developing countries in series, such as in Huang et al. in 2013 (12) and in Kumar et al. in 2022 (8), which is associated with a larger tumor size, longer duration of symptoms, the presence of hydrocephalus, and delayed access to neurosurgical evaluation. In one study (8), the rate of postoperative visual improvement decreased with higher rates of visual loss upon presentation (66% of WHO category B improved, but only 27% of WHO category C and 11% of WHO category D) (8). This could imply that a) visual impairment is probably overlooked in VS and that b) this delay in detection and treatment affects a patient’s odds of regaining sight.

Rarer still is the association between VS and visual loss caused by prolonged papilledema in the absence of hydrocephalus, as described in several case reports (3, 9, 11, 13–16). In 2013, Kumar et al. (9) studied a group of such patients with large VS (*n* = 23) and found that the mean cisterna magna CSF protein (CMCP) levels were higher in patients with VS than in controls, that the mean CMCP levels in patients with visual loss were higher than those without visual loss (including 16 patients with and seven patients without papilledema), and that both the shorter duration of symptoms and the total tumor volume correlated with higher CMCP levels (*p* < 0.04, multivariate analysis). The authors claimed that microcystic degeneration or intratumoral necrosis is the source of hyperproteinorrachia, features associated with a rapidly growing tumor (9). No formal explanation on those without hydrocephalus was given at the time; however, other similar case reports have been found in the literature (3). **Table 2** summarizes the different published case reports.

**Table 2:** Summary of the published case reports and case series of vestibular schwannoma- and papilledema-related visual loss in the absence of hydrocephalus.

Author, year	Study type	Clinical scenario	Author’s discussion
Kumar et al., 2016 (9)	Case series	40 patients with VS were analyzed, finding a statistically significant difference (*p* < 0.001) in the cisterna magna CSF protein levels in VS patients with vision loss (561.4 + 186.9 mg/dl) compared with those without visual loss (314.2 + 160.9 mg/dl). The grade of visual diminution was proportional to the mean CMCP levels (*p* < 0.001).	Hyperproteinorrachia seemingly is caused by tumoral degeneration and necrosis from neoplastic growth exceeding blood supply, according to the authors. This series shows a statistically significant relationship between hyperproteinorrachia and visual loss in patients with VS.
Candanedo, et al., 2017 (13)	Case report	A 20-year-old with bilateral papilledema, with a 40-mm VS, and no hydrocephalus, but with an enlarged optic nerve sheath on MRI. Postoperatively improved visual acuity, with no papilledema on follow-up.	Bilateral papilledema may have preceded ventricular dilation, with no other sign of increased ICP. The authors quote other reports suggesting that protein secretion into the CSF may have impaired CSF reabsorption.
Harada, et al., 1998 (16)	Case report	A 26-year-old with NF2 presenting with bilateral VS and progressive bilateral visual loss. Severe papilledema was found with no evidence of hydrocephalus.	Gavotto et al. suggest intermittent obstruction of the ventricular system as a possible but weak explanation of the absence of hydrocephalus. Impaired CSF absorption from elevated CSF protein levels is also presented in this article as a theory. Visual function after resection did not improve, as the optic atrophy upon diagnosis was already severe. Intracranial pressure was not estimable preoperatively due to the risk of herniation from lumbar puncture.
Gavotto et al., 2022 (14)	Case report	A 39-year-old with optical coherence tomography-confirmed bilateral papilledema, enlarged blind spots, and a 26-mm × 21-mm right VS, with no hydrocephalus, and hyperproteinorrachia. Postoperative follow-up showed progressive visual improvement, with resolution of papilledema at 3 months.	The authors propose hyperproteinorrachia blocking the CSF resorption capacity of the optic nerve (ON) sheaths, causing “local hypertension” of the ON, manifested as papilledema. They postulate tumoral TNF alpha-related secretion as the cause of ON damage.
Bloch et al., 2003 (3)	Case series	Five patients with VS and one patient with trigeminal schwannoma, presenting with non-obstructive hydrocephalus and hyperproteinorrachia. Post-op showed reduction in CSF protein levels.	The discussion suggests a saturation of pacchionian granulations, altering the CSF flow into the venous system. Fibrinogen from chronic inflammation and possibly tumoral bleeding are proposed as the cause of CSF outflow resistance and of non-obstructive hydrocephalus.
Grainger et al., 2005 (11)	Case report	A 56-year-old presenting with bilateral diminished visual acuity. CT revealed VS, with a normal ventricle size. Noninvasive ICP monitoring showed no intracranial hypertension. Cisternal CSF showed hyperproteinorrachia.	The causes of optic disc edema are unclear, especially in the absence of hydrocephalus, cranial hypertension, and other normal pressure hydrocephalus signs. Grainger et al. suggest high CSF protein levels as the cause, but do not offer insights as to the mechanisms involved.
Matos et al., 2016 (15)	Case report	A 64-year-old presenting with severe bilateral disc edema and retinal hemorrhage. Upon undergoing imaging was found to have a right CP angle lesion, with no obstructive hydrocephalus. At 10 months post-op, a marked regression of papilledema was found.	Hyperproteinorrachia causing CSF stasis is proposed as a mechanism for papilledema without hydrocephalus. However, CSF analysis was not made. The improved visual outcomes after surgery, however, seem to strengthen the association.

*VS*, vestibular schwannoma; *CSF*, cerebrospinal fluid; *CMCP*, cisterna magna CSF protein; *ICP*, intracranial pressure; *CP angle*, cerebellopontine angle.

Evidence suggests that VS present a complex tumoral microenvironment with significant immune cell infiltration, the extent of which has been shown to correlate both histologically with tumor growth and angiogenesis (17) and clinically with the degree of invasion (7). The upregulation of pro-inflammatory cytokines, including IL-1β, IL-6, and TNF-α, within intratumoral VS samples (17) could explain the presence of the high CSF protein in some cases, although the exact composition is not clear at this time.

The ON is a complex structure that originates from retinal ganglion cells, which then leaves the optic globe, crosses the orbit and optic canal, and finally enters the intracranial subarachnoid space, where it forms the optic chiasm. The axons of the ON are enveloped by all three layers of meninges after the lamina cribrosa, creating a subarachnoid space that extends through the intraorbitary and intracanalicular portions; thereafter, it connects with the intracranial subarachnoid space, thus allowing CSF flow through these two compartments (4). It is worth noting that the ON subarachnoid space behaves as a sort of reservoir due to its blind pouch structure at the ocular distal end (limited by the lamina cribrosa) and a similar phenomenon at the very narrow proximal optic canal. The subarachnoid CSF flow appears to be mostly one-directional, entering from the intracranial compartment through a greater volume gradient with each arterial pulsation, which begs the question of how this CSF is eventually reabsorbed (4). Although no single mechanism appears to explain CSF outflow, the glymphatic system appears to be involved. In this regard, papilledema has long been specified to be a consequence of an increased intracranial pressure (18) (and rarely as a manifestation of altered local ON CSF dynamics).

Both the brain and the retina display intense metabolic activity; as such, they require a special system that clears chemicals and ions between fluid spaces and thus creates a stable environment for synaptic transmission. Contrary to peripheral organs, however, the central nervous system (CNS) does not have a classic lymphatic system, partly due to the existence of the blood–brain barrier, which has long since been regarded as an “immunologically privileged site.” In 2012, Iliff et al. (19) described a paravascular glial-associated lymphatic (or “glymphatic”) system that has since become an active area of research (20, 21). The glymphatic system consists of three compartments among which water, ions, and other molecules are moved through and out of the CNS (5). The CSF circulating the subarachnoid space initially enters the perivascular Virchow–Robin space deep into the brain parenchyma, where water moves between the CSF and the interstitial fluid (ISF) at astrocyte end-feet channels surrounding the capillaries. Metabolic waste is eventually led out through perivenous spaces and out of the CNS either via the meningeal lymphatic system found in dural sinuses or back to the subarachnoid space. The exchange of water molecules is driven by aquaporins, which are transmembrane channel proteins that are widely distributed in the human body. Aquaporin-4 (AQP-4) is the most abundant of the 13 subtypes in the brain and cranial nerves, which expedites the outflow of water from the ISF (brain parenchyma) to the CSF (subarachnoid space) (5). APQ-4 has also been observed to be highly abundant in the ON (6).

The CSF flow has been observed to be impaired in different states of neuroinflammation, mainly in traumatic brain injury, stroke, neuroinfection, and neurodegenerative diseases (e.g., Alzheimer’s and Parkinson’s disease) (5). This is a consequence of the effect of inflammatory mediators in the glymphatic system, including reactive astrocyte changes in its morphology, blood–brain barrier opening, and altered AQP-4 expression, among others. AQP-4 channel expression increases in astrocytes; however, contrary to functional channels that are inserted along the perivascular end-feet where the CSF–ISF exchange occurs, these insert themselves in the cell body and the perisynaptic region, which ultimately leads to a decreased CSF–ISF exchange and an overall decreased glymphatic flow, with a resultant increase in the subarachnoid compartment (5).

Based on these findings from the literature, we believe it is possible that impairment of the glymphatic system by affection of the ON AQP-4 expression in the context of intense VS tumoral neuroinflammation is responsible for the association between ON papilledema without hydrocephalus, hyperproteinorrachia, and VS. However, it is necessary to perform a larger observational clinical study, as well as biological experimental analyses, to reinforce the strength of this association.

This case report presents an unusual clinical scenario with profound objective clinical deficits in a patient (campimetry-proven bilateral visual loss) with an updated understanding of the CSF physiology currently available in the literature. Further observational and laboratory research is needed to strengthen this clinical association and to demonstrate biological plausibility.

## Conclusion

In summary, papilledema without hydrocephalus is a rare finding in VS whose pathophysiology warrants more research, both a) observational to assess the true strength of this association and the frequency with which it occurs and b) in the laboratory to determine the nature of this phenomenon.

We present a case of bilateral papilledema with visual function decline with no extrinsic tumoral compression of the visual pathway or hydrocephalus. Through a literature review of visual impairment in VS, in the terms of CSF dynamics, we hypothesized an altered glymphatics via microenvironment-induced changes in AQP-4 function.

The ON subarachnoid space is a relatively “isolated” compartment from the rest of the ventricular system, which receives CSF flow from the intracranial compartment, but whose outflow is anatomically restricted and must therefore rely on other mechanisms to clear retinal metabolites and water from the ISF and CSF. AQ-4 is a transmembrane channel protein involved in the glymphatic system that is shown to be abundant in this area.

This case report and previous small series, taken into account with the increasingly complex studies on the CSF dynamics, should prompt clinicians to consider alternative explanations for neurological deficits in the absence of other findings, such as preoperative imaging or intraoperative findings. Bigger case series and biological research are necessary to study such phenomena.

## Data Availability

The original contributions presented in the study are included in the article/supplementary material. Further inquiries can be directed to the corresponding author.
